# Individual Changes in Respiratory Compliance Upon Immersion May Predict Susceptibility to Immersion Pulmonary Edema

**DOI:** 10.1186/s40798-023-00590-8

**Published:** 2023-06-01

**Authors:** Olivier Castagna, Arnaud Druelle, Guillaume Michoud, Thibaut Prevautel, Jean-René Lacour

**Affiliations:** 1 Underwater Research Team – ERRSO, Military Biomedical Research Institute-IRBA, Toulon, France; 2 LAMHESS (UPR 6312), Université de Nice, Nice, France; 3 French Navy Diving School, St Mandrier, France; 4 2nd Regiment Etranger de Parachutistes, Calvi, France; 5 Department of Cardiology, Laveran Military Hospital (HIA Laveran), Marseille, France; 6 Université Jean Monnet, 42000 Ste Etienne, France

**Keywords:** Immersion pulmonary edema, Pulmonary compliance, Work ok breathing, Individual susceptibility

## Abstract

**Background:**

Immersion pulmonary edema (IPE) is a frequent diving accident, and it is the primary cause of hospitalization for young military divers during training. The objective of this study was to identify immersion-induced parameters predicting individual susceptibility to IPE.

**Methods:**

Eighteen experienced male divers having completed at least 100 dives were recruited. Eight divers had previously been hospitalized for IPE (IPE), and the other ten had never developed IPE (non-IPE). The two groups were matched for age, BMI, and number of dives performed. Ventilatory function and overall compliance of the respiratory system (Crs) were measured on land and during head-out-of-water immersion. Subjects also performed 30 min of fin swimming in a channel at 33 m min^−1^. Following this exercise, the presence of extravascular lung water, revealed by ultrasound lung comets (ULC), was assessed.

**Results:**

In the whole group, the decrease in Crs upon immersion correlated with the immersion-induced alterations to expiratory reserve volume, ERV (*r*^2^ = 0.91; *p* < 0.001), inspiratory reserve volume, IRV (*r*^2^ = 0.94; *p* < 0.001), and tidal volume, Vt, changes (*r*^2^ = 0.43; *p* < 0.003). The number of ULC correlated strongly with immersion-induced changes in ventilatory function (*r*^2^ = 0.818; *p* < 0.001 for ERV, *r*^2^ = 0.849; *p* < 0.001 for IRV, *r*^2^ = 0.304; *p* = 0.0164 for Vt) and reduced Crs (*r*^2^ = 0.19; *p* < 0.001).

The variations of ERV, IRV, and Crs at rest induced by head-out-of-water immersion and the number of ULC measured after swimming for 30 min were significantly greater in IPE subjects.

**Conclusion:**

In the face of similar immersion stresses, the extent of alterations to ventilatory function and the number of ULCs were very different between individuals but remained statistically correlated. These parameters were significantly greater in divers with a history of IPE. Alterations to pulmonary function and, in particular, to pulmonary compliance induced by head-out-of-water immersion, through their effects on work of breathing appear to allow the identification of divers with a greater susceptibility to developing IPE. Measurement of these parameters could therefore be proposed as a predictive test for the risk of developing IPE.

**Supplementary Information:**

The online version contains supplementary material available at 10.1186/s40798-023-00590-8.

## Key Points

- Immersion Pulmonary Edema (IPE) can occur in highly fit individuals, such as military divers and triathletes, and is the leading cause of hospitalization among young military divers during training.
- To preserve the health of young divers, predictive tests of individual susceptibility to IPE should be proposed, similar to those proposed for High Altitude Pulmonary Edema (HAPE).
- Our findings confirm the fundamental role of immersion-induced changes in lung function in the development of IPE. Alterations in pulmonary function, particularly pulmonary compliance, appear to allow for identification of divers with a higher susceptibility to developing IPE.

**Supplementary Information:**

The online version contains supplementary material available at 10.1186/s40798-023-00590-8.

## Background

Immersion pulmonary edema (IPE) consists of an increase in transmural pulmonary capillary pressure that induces transudation into interstitial tissue. [1]. IPE causes dyspnea while swimming, scuba, or breath-hold diving, and may be accompanied by cough, hemoptysis, and severe hypoxemia; it can even lead to death [2–5]. IPE is a non-trivial phenomenon for emergency physicians, even though rest and normobaric oxygen therapy usually produce rapid relief of symptoms without sequelae [6]. Death is generally due to drowning and/or reversible myocardial dysfunction [7]. Factors predisposing to the development of IPE include age over 50 years, cardiovascular risk factors, and/or high blood pressure [8–10]. However, the condition also occurs in very fit people, such as military divers and triathletes. [4, 11–13]. Thus, in our hyperbaric medicine department, IPE has become the primary cause of hospitalization of young military divers during training.

The accumulation of fluid in the lungs can extend to the alveolar air spaces [14], leading to the appearance of Ultrasound Lung Comets (ULC) [15]. The number of ULC is an indication of the extent of accumulation of extravascular lung water (EVLW). In healthy young divers exposed to similar immersion constraints (same depth and duration of immersion, swimming speed and breathing apparatus), some subjects systematically present a significantly higher number of ULC. The concept of individual susceptibility to Immersion Pulmonary Edema (IPE) has been suggested [6]. Wilmhurst et al. [16, 17] conducted a study examining this susceptibility and its association with vascular responses in individuals undergoing cold water immersion. They observed that individuals prone to IPE exhibited a greater increase in forearm vascular resistance compared to control subjects when their head and neck were exposed to ice-cold water. These findings suggest that abnormal vascular responses, particularly vasoconstriction, may contribute to the development of IPE in susceptible individuals. The heightened vasoconstrictive response indicated by the increased vascular resistance in the forearm has the potential to result in elevated pulmonary capillary pressures and subsequent pulmonary edema.

Research has suggested that certain genetic variants, such as polymorphisms in genes involved in the regulation of inflammation and vascular permeability, may influence the individual response to immersion and increase the risk of IPE [18]. In previous studies, young, healthy professional scuba divers finning exercises at shallow depth were found to have widely different ULC scores [19, 20]. Because IPE presents an immediate risk of drowning but also of myocardial dysfunction, with a risk of recurrence estimated by Gempp et al. [8] at 15%, military divers having developed IPE are declared unfit—at least temporarily—contributing to the attrition of military divers.

Both IPE and High-Altitude Pulmonary Edema (HAPE) share similar underlying mechanisms, including increased pulmonary capillary pressure, alterations in vascular permeability, and inflammatory responses, all of which can lead to the accumulation of fluid in the lungs [21–23]. Additionally, individual predispositions can increase the risk of developing potentially life-threatening HAPE [24–27]. Given the difficulty in predicting HAPE, especially in individuals without prior high-altitude exposure, various altitude simulation tests have been developed and validated [28, 29]. It seems appropriate to initiate a similar approach for IPE to identify divers who have a high susceptibility to IPE occurrence.

Immersion modifies pulmonary function, leading to a decrease in expiratory reserve volume (ERV), forced vital capacity (FVC) [30, 31], and overall compliance of the respiratory system (Crs) [32–34]. This immersion-induced alteration of the Crs is not without consequence, as it is associated with an increase in the work of breathing (WOB) both on land [35, 36] and during immersion [30]. Retrospective analysis of individual ventilatory function parameters, and how they vary upon immersion, shows extensive interindividual variability [33, 34].

WOB plays a fundamental role in the occurrence of IPE, as inspiratory work specifically promotes the accumulation of EVLW both on land [35, 36] and during immersion [30]. An increase in WOB leads to increased transmural hydrostatic pressure differences between the lumen of the lung capillaries and the interstitial fluid in bronchial bundles and alveoli [9], alters cardiac function [37], and can trigger acute pulmonary edema [3]. Significantly, WOB is greater during immersion than on land, because the external hydrostatic pressure creates a greater transpulmonary pressure difference [12, 38–41]. With identical immersion constraints, WOB values are extremely variable between individuals [41], although they remain strongly correlated with the number of ULCs. This dispersion of WOB values in young, healthy subjects remains unexplained.

Understanding the causes of interindividual variability in WOB during immersion is crucial to identify the pathophysiological mechanisms of IPE, determine its causes, and identify individuals at higher risk. Given the challenges of measuring individual WOB during immersion, we sought a more accessible ventilatory test to assess susceptibility to IPE.

The main objective of this study was to determine whether the changes in lung function and, in particular, lung compliance induced by head-out-of-water immersion are significantly different between individuals who had previously developed IPE and individuals who had never experienced IPE.

This was done with the intention of potentially suggesting the use of pulmonary function measurement as a means of identifying individuals who are more susceptible to IPE.

## Methods

### Subjects

**Fig. 1:**
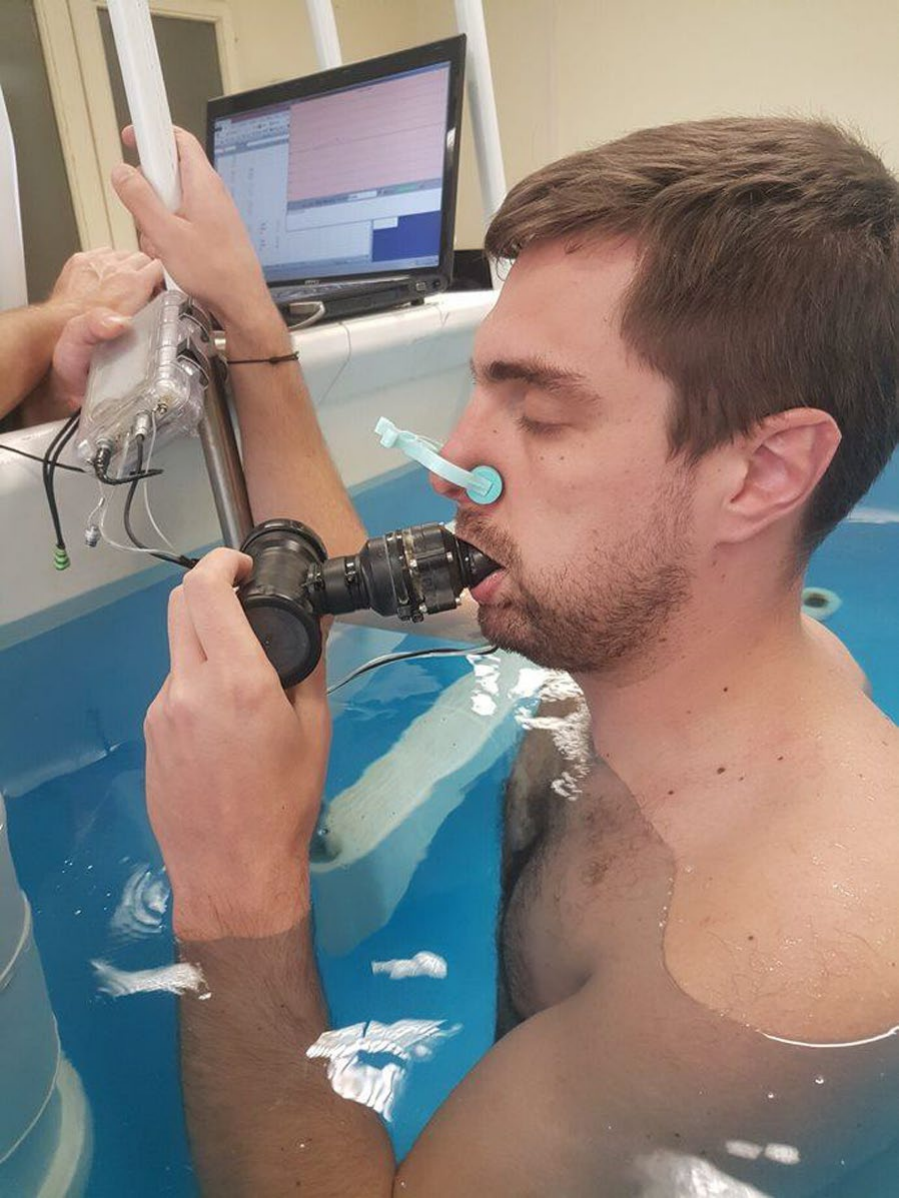
A subject during measurement of immersed static respiratory compliance. The subject is kneeling upright and wearing a nose clip. The water comes up to the subject’s sternal notch. A pressure sensor is integrated in the mouthpiece, which is connected through a pipe to the calibrated syringe injecting or sucking up the preset volume

Eighteen male volunteers were enrolled in the study. All subjects were healthy non-smokers without known pulmonary or cardiovascular disease or symptoms. Subjects gave their written informed consent for participation in the study. All the experimental procedures were conducted in line with the declaration of Helsinki, and the study protocol was approved by the local ethics committee (Comité de Protection des Personnes « ile de France II» n°: 21.05.05.35821 RIPH1 HPS; N° ID RCB: 2021-A01225-36). The diver in the photograph in Fig. 1 gave his written consent for use of this image.

Participants were assigned to two groups. A control group composed of ten experienced divers having already performed at least 100 dives without ever having presented an IPE (non-IPE), and a group of eight divers who had previously been hospitalized for IPE (IPE). The two groups were matched for age, BMI and number of dives performed (Table 1).

**Table 1:** Morphological characteristics of the subjects in both populations

	Age (years)		Height (cm)		Weight (kg)		BMI (kg m^−2^)	
	Non-IPE	IPE	Non-IPE	IPE	Non-IPE	IPE	Non-IPE	IPE
Median	32.3	35.5	178.3	181.5	76.4	76.1	24.1	23.8
Mean	32.5	35.9	178.6	181.9	76.5	76.9	24.2	23.7
SD	6.35	7.63	6.59	6.52	6.47	6.37	3.98	3.82
*p*	n.s		n.s		n.s		n.s	

The control group of divers (non-IPE) had never developed an immersion pulmonary edema (IPE). Divers in the IPE group had previously been hospitalized for an immersion pulmonary edema. In BMI, body mass index, statistical significance was determined using the Mann–Whitney test for unpaired data

### Study Design

A series of measurements were taken for each subject in the following order:

1.A lung ultrasound at rest on land, to verify the absence of ULC,

2.Analysis of ventilatory function and measurement of Crs at rest on land,

The subject was then immersed, head above water, in an experimental pool, and

3.Ventilatory function and Crs were once again measured while immersed,

4.The subject then performed a swimming exercise in the "swim flume" for 30 min,

5.At the end of the exercise, a second lung ultrasound was performed to count the number of ULCs present.

The air temperature in the laboratory and the water temperature in the swim flume were both maintained at 27 °C. Each subject performed all the tests within a single day in the IRBA Physiology Lab.

#### Ultrasound Lung Comets (ULC) Assessment

Ultrasonographic examinations were performed by an experienced sonographer using a commercially available ultrasound system (Vivid I; GE Médical, Horten, Norway) with a 1.5- to 4-MHz phased array transducer. The presence of EVLW was assessed on lung ultrasounds by counting the number of B-lines or ULC [42].

#### Spirometric Measurements

A Cosmed Quark PFT Ergo device (Cosmed, Rome, Italy) was used to assess the slow vital capacity (VC), expiratory (ERV) and inspiratory reserve volume (IRV), and tidal volume (Vt), according to the guidelines published by the American Thoracic Society and the European Respiratory Society [43]. Each subject repeated the spirometric maneuver five times in each condition. For each condition, the two extreme values were discarded and the mean of the remaining three was retained.

#### Static Compliance of the Respiratory System (Crs)

Crs was measured in the following two conditions: upright, standing on land (out of the water) as control condition, and upright, on the knees immersed up to the sternal notch as immersed condition (head-out-of-water). Tests were performed with the subject wearing a nose clip and breathing only through the mouthpiece. A subject during measurement of immersed static respiratory compliance is presented in Fig. 1.

Respiratory compliance was determined by replicating the protocols previously described [33, 40]. For each subject, a static pressure–volume curve was constructed in each of the four conditions using values obtained thanks to a specific device, designed in house [40, 41]. The device contains a pressure sensor (MPXV70007DP Freescale), with a ± 70-mbar measurement range, placed immediately behind a mouthpiece, followed by a flowmeter and a three-way valve. Depending on the setting of the three-way valve, the subject, wearing a nose clip, inspired air either from the room or from a syringe filled with one of the following preset volumes: 0.2, 0.5, 1, 1.5, 2, 2.5 and 3 L.

For the measurement cycle, the subject initially breathed ambient air freely. At the expiratory end of a quiet tidal volume cycle (i.e., a relaxation volume held without any effort and nominated as *V*_*relax*_), the valve was closed and the subject remained apneic with an open glottis. This relaxed volume was similar to that described by Taylor and Morrison [33]. The mouth pressure in these conditions should be 0 mbar (equal to ambient barometric pressure). If this was not the case, the subject was asked to repeat the maneuver. The pulmonary gas volume corresponds to the spontaneous relaxation volume in each condition. In the Up-Air condition, this relaxation volume corresponds to the forced residual capacity (FRC). Once this parameter had been measured, the valve was opened toward the syringe and the subject inspired the preset air volume. As soon as the syringe was empty, the valve was closed, and the subject remained apneic with an open glottis for 4–6 s during which time the airway pressure was recorded. After each inspired volume, the airway pressure was negative (lower than the ambient atmospheric pressure). The maneuver was performed in a similar manner with the subject starting from *V*_*relax*_ to exhale a preset volume (i.e., syringe plunger set and expiration starting with an empty syringe). The volumes to be exhaled were as follows: 0.1; 0.2; 0.5; 1; 1.5; 2; 2.5 and 3 L. As soon as the plunger reached the preset stop, the valve was closed and the airway pressure was recorded during the 4–6 s apnea with open glottis. After each expired volume, the pressures recorded were positive (i.e., higher than atmospheric) and reflected the transpulmonary pressure. Subjects were trained to perform the maneuvers so as to reproduce the relaxation volume and the open glottis apnea for each preset volume (i.e., the syringe content or the preset maximal filling allowed). Training required from 30 to 60 min, depending on the subject. Each airway pressure measurement with open glottis at each inspired and expired volume was repeated five times. The two extreme values were discarded before averaging the three remaining values. When the subject could not successfully complete the maneuvers despite several training cycles, their data were excluded from the study.

Four curves, one in each condition, were constructed for each subject. Curves had an average of 32 ± 8 points depending on the number of volume points achieved. Each curve was fitted using a second square polynomial regression:

$$P = aV^{2} - bV - c$$

where *P* is the pressure measured, and *V* is the gas volume inspired or expired from *V*_relax_. All volumes were corrected for body temperature and partial pressure of water.

Each curve was characterized by a value representing compliance of the respiratory system (i.e., combining lung + chest wall, Crs), which corresponds to the regression slope for the 1-L increase in volume above *V*_relax_ (in the relevant condition) relative to the pressure increase associated with this 1-L volume (Crs = ∆*V*/∆*P*).

#### Fin Swimming Exercise

All fin swimming exercises were conducted in a motorized swimming flume (Endless Pools, Dilsen-Stokkem, Belgium) located within the IRBA Physiology Lab. The linearity and the relationship between engine power and water speed were fully calibrated using a water flowmeter. Subjects performed a 30-min fin swimming exercise at a constant flume speed of 33 m min^−1^. This speed corresponds to the fin swimming speed required of military divers in training and during missions.

#### Statistics

Data were statistically analyzed using Prism 6 software (GraphPad Software, La Jolla, California). Data distribution was assessed using a Kolmogorov–Smirnov test. Normally distributed values recorded at two different times or between both populations were compared using the Student *t* test for paired data; Wilcoxon’s paired signed rank test was used for non-normally distributed data. Correlations between parameters were assessed using the Pearson's test or Spearman’s test (depending on whether values were normally distributed). Differences were considered statistically significant at *p* < 0.05. All values are expressed as median, 25–75% interquartile, mean, ± SD.

## Results

Morphological characteristics of the subjects are presented in Table 1. No differences in age, height, body mass, and BMI were observed between the two groups of subjects.

For the whole study population, immersion-induced changes in spirometric parameters (VC, Vt, ERV, and IRV), and Crs and the number of ULC measured in all divers at the end of the fin swimming exercise are reported in Table 2. Values for individual subjects can be found in Additional file 1.

**Table 2:** Changes to spirometric data (VC, Vt, ERV, and IRV), and Crs induced by head-out-of-water immersion, and number of ULCs measured at the end of fin swimming exercise for the whole study population

	∆VC (L)	∆ERV (L)	∆IRV (L)	∆Vt (L)	∆Crs (L/kPa)	ULC
Median	− 0.44	− 1.13	0.49	0.20	0.93	16.5
25%/75% percentile	− 0.50/− 0.41	− 1.52 /− 0.8	0.14 /0.87	0.16 /0.22	0.70 / 1.36	7.2 / 24.2
Mean	− 0.45	− 1.19	0.54	0.20	1.01	15.9
S.D	0.06	0.46	0.45	0.03	0.46	11.3
Variation	↘9%	↘77%	↗40%	↗18%	↘85%	–
Statistical significance	*p* < 0.0001	*p* < 0.0001	*p* = 0.0002	*p* < 0.0001	*p* < 0.0001	–

Changes to spirometric data (VC, Vt, ERV, and IRV), and Crs induced by head-out-of-water immersion, and number of ULCs measured at the end of fin swimming exercise for the whole study population. Individual subject values are available in Additional file 1. Median values are shown, along with 25% /75% percentile, mean and standard deviation. Data variation is also expressed as a percentage (%). Statistical significance was determined using the Student’s t test for paired data when data were normally distributed, or Wilcoxon’s paired signed rank test for non-normally distributed data

Vital capacity (VC); expiratory reserve volume (ERV); inspiratory reserve volume (IRV); tidal volume (Vt); ultrasound lung comets (ULC)

### Immersion‑Linked Changes to Spirometric Lung Volumes

Head-out-of-water immersion was accompanied at rest by a collapse of ERV (− 77%; *p* < 0.0001) that was not totally compensated by the increase in IRV (+ 40%; *p* = 0.0002). Consequently, the slow VC was significantly decreased (− 9%; *p* < 0.0001), and mean Crs values dropped by 85% (*p* < 0.0001). Following the fin swimming exercise, the number of ULC averaged 15.9, with a very high interindividual variability. No ULC were observed in any subject before immersion.

In the whole group, the decrease in Crs upon immersion correlated with the immersion-induced alterations to ERV (*r*^2^ = 0.91; *p* < 0.001), IRV (*r*^2^ = 0.94; *p* < 0.001), and Vt changes (*r*^2^ = 0.43; *p* < 0.003) (Table 3). The Crs values were not related to the VC alterations.

**Table 3:** Correlation between changes in Crs and changes in spirometric data (VC, ERV, IRV, and Vt) induced by head-out-of-water immersion

	∆ Crs (L/kPa) vs. ∆ VC (L)	∆ Crs (L/kPa) vs. ∆ ERV (L)	∆ Crs (L/kPa) vs. ∆ IRV (L)	∆ Crs (L/kPa) vs. ∆ Vt (L)
*r*^2^	0.1164	0.9131	0.94	0.4269
*p*	0.1659	< 0.0001	< 0.0001	0.0033

Correlation between changes in Crs and changes in spirometric data (VC, ERV, IRV, and Vt) induced by head-out-of-water immersion. Individual subject values are available in Additional file 1. Statistical significance was determined using Spearman’s test

Compliance of the overall respiratory system (combined lung and chest wall) (Crs); vital capacity (VC); expiratory reserve volume (ERV); inspiratory reserve volume (IRV); tidal volume (Vt); ultrasound lung comets (ULC)

**Fig. 2:**
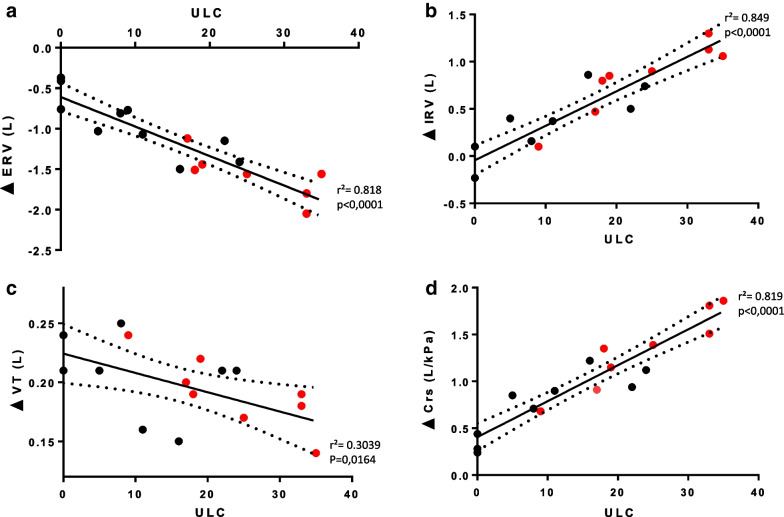
Correlation between number of ULCs measured at the end of fin swimming exercise and spirometry data (VT, ERV, and IRV), and Crs induced by head-out-of-water immersion. Correlation between ULCs measured at the end of the fin swimming exercise and spirometry data, and compliance of the respiratory system induced by head-out-of-water immersion. **a** Correlation between ULCs and variation of ERV. **b** Correlation between ULCs and variation of IRV. **c** Correlation between ULCs and variation of Vt. **d** Correlation between ULCs and variation of Crs. Ultrasound lung comets (ULC); expiratory reserve volume (ERV); inspiratory reserve volume (IRV); tidal volume (Vt); compliance of the overall respiratory system (combined lung and chest wall) (Crs). Each point corresponds to the values for an individual subject. Divers in the non-IPE group are represented by black dots (*n* = 10); divers in the IPE group are represented by red dots (*n* = 8). *r*^2^ and statistical significance was determined using Spearman’s test

The number of ULC observed at the end of the fin swimming exercise was not correlated with the variation in VC induced by immersion, but was weakly correlated with Vt variations, and very strongly with variations in ERV and IRV (Fig. 2). The changes in Crs induced by head-out-of-water immersion at rest very strongly correlated with the number of ULC observed at the end of the fin swimming exercise (Fig. 2).

**Fig. 3:**
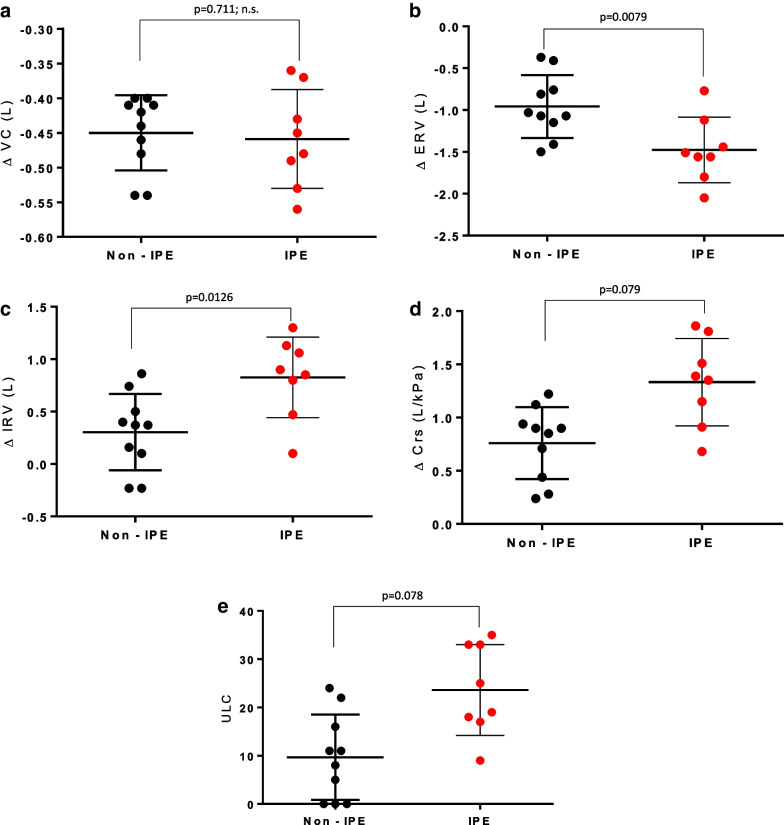
Changes to VC, ERV, IRV, and Crs induced by head-out-of-water immersion and ULC observed after fin swimming exercise in both populations (non-IPE vs. IPE). Changes to VC, ERV, IRV and Crs induced by head-out-of-water immersion and ULC observed after fin swimming exercise in both populations (non-IPE vs. IPE). Each point corresponds to the value observed for an individual subject. Divers in the non-IPE group are represented by black dots (*n* = 10); the red dots correspond to divers in the IPE group (*n* = 8). Statistical significance was determined using a Mann–Whitney test. Compliance of the overall respiratory system (combined lung and chest wall) (Crs); vital capacity (VC); expiratory reserve volume (ERV); inspiratory reserve volume (IRV); tidal volume (Vt); ultrasound lung comets (ULC)

The variations of ERV, IRV, and Crs at rest induced by head-out-of-water immersion and the number of ULC measured after swimming for 30 min were significantly greater in IPE subjects (Fig. 3). No significant difference in VC values was observed between the two groups.

## Discussion

To the best of our knowledge, this is the first study to investigate the impact of pulmonary function on IPE occurrence.

In our study, we found that individuals who had previously experienced Immersion Pulmonary Edema (IPE) showed significantly greater impairment in lung function, specifically lung compliance, compared to those without a history of IPE. By quantifying and comparing these alterations, we aimed to identify markers that can accurately predict the risk of IPE occurrence.

Firstly, we delved into the step-by-step analysis of the alterations in ventilatory function, starting with the simplest changes and progressing toward the examination of pulmonary compliance. Secondly, we explored the reasons why these alterations in ventilatory function were closely linked to the development of IPE. Lastly, we addressed the possibility of utilizing pulmonary function analysis as a marker for assessing the risk of IPE occurrence.

### Effect of Immersion on Lung Volumes

The effects of immersion on lung volumes have been well documented in previous studies [30–32, 44–47]. Immersion and lying supine both result in a cephalad displacement of the diaphragm and a subsequent reduction in lung volumes [30, 48]. Our findings of an increased IRV and decreased ERV during immersion are consistent with the results reported by Paton and Dahlbäck [30, 31]. Furthermore, intrathoracic pooling of blood volume has been shown to contribute to the decrease in lung gas volume [31, 49, 50].

### Effect of Immersion on Lung Compliance

The amplitude of the decrease in overall Crs induced by head-out-of-water immersion reported here was in agreement with data in the literature [32, 33, 40].

This decrease in Crs is the result of a combination of three factors: first, the decreased compliance of pulmonary parenchyma, related to the increase in lung blood content induced by immersion [31, 50–52], second, the decrease in lung volume induced by water immersion [53], and third, the increased chest wall elasticity, which plays a substantial role in the reduction in lung volume upon immersion[33]. The contribution of each component probably varied between subjects [44, 45]; it thus remains impossible to circumscribe this parameter based solely on the measurements presented here. However, the strong correlation between the individual decreases in ERV and Crs values suggests that the reduction in lung volume upon immersion may play a significant role.

Furthermore, immersion causes pulmonary blood flow redistribution, but also enlarges VA’/Q’ disparities relative to the increasingly uneven distribution of alveolar ventilation and to gas trapping in the airways [31, 48]. Strong indirect evidence indicates that immersion induces similar changes in cardiac and pulmonary blood volume [49].

The characteristics of the chest walls and lung parenchyma are highly variable between individuals. This probably explains why the magnitude of the alterations in Crs induced by immersion was so large between subjects (min-max, 0.28–1.86 L/kPa).

### Effects of Crs Impairment on WOB and IPE Occurrence

Crs impairment results in increased WOB both on land [35, 36] and during immersion [30]. In response to decreased Crs, enhanced transthoracic depressions are required to mobilize gas, resulting in increased WOB. This increased WOB favors the occurrence of acute lung edema.

Acute pulmonary edema due to increased WOB may develop on land, in healthy subjects, in response to a high inspiratory effort and the resulting large negative intrathoracic and alveolar pressures [37]. An increased negative intrathoracic pressure induces dilation of both the right atrium and the right ventricle, resulting in a drop in right heart pressure. The ensuing increase in the pressure gradient between the vena cava and the right atrium creates an increase in venous return to the right heart [54]. Right ventricular contractility is thus improved thanks to the Frank-Starling mechanism [55]. The combination of this higher hydrostatic pressure in the pulmonary capillary and the lower interstitial pressure in the lung promotes extravasation of fluid from the plasma, initially into interstitial tissues and then across the alveolar membrane into the alveolar air space [56].

During diving, the mechanisms linking WOB and IPE occurrence have already been described [12, 19, 41]: each tidal inspiration requires a greater WOB than on land, achieved through substantial lowering of thoracic, airway and mouth pressures. This increased negative airway pressure enhances fluid extravasation from the pulmonary capillaries [9].

As there is a very large interindividual variation in the alteration of Crs induced by immersion [30, 33, 34, 40], it is not surprising to observe a strong variation in WOB between subjects in the same immersion conditions.

### Perspectives

Due to its high rate of recurrence, military divers who have been hospitalized for IPE are declared medically unfit for further scuba diving.

The correlation between lung function alterations induced by head-out-of-water immersion and the number of ULC described in this article is statistically robust. Furthermore, we observed significantly lower alterations to lung function and ULC counts in non-IPE divers. Therefore, it can be hypothesized that pulmonary function measurements during immersion can predict the number of ULCs that will be observed following an apnea exercise.

Measurement of immersion-induced alterations to ventilatory function and Crs could be a good predictive test for the development of IPE. Performing these measurements could help detect subjects with a greater individual susceptibility to IPE.

Future studies, including a larger number of divers, should allow us to define the threshold Crs value beyond which the risk of occurrence of IPE increases significantly.

### Limitations

To explain the observed link between the decrease in Crs and the number of ULC, (via the increase in WOB), we relied on previous work. However, in the present study, we did not actually verify this parameter. A future study combining joint measurements of lung compliance, WOB, and the number of ULC will allow us to confirm this hypothesis.

We also note that Crs values measured simply on land did not correlate with the number of ULCs (*p* = 0.265). Only the head-out-of-water-induced decrease in Crs was. Thus, measurement of Crs on land cannot be used to predict susceptibility to ULC. It is necessary to analyze the changes in pulmonary function induced by immersion and, in particular, the alteration of Crs.

In addition, we divided the subjects in our study into two groups (non-IPE *vs*. IPE). Although we were careful to include only experienced divers with at least 100 dives, it is not possible to certify that within the non-IPE group, some subjects will not go on to develop a true IPE. Therefore, the separation of our population into two groups was relatively artificial.

The study population was relatively small (18 divers); consequently, any conclusions drawn from the results will need to be confirmed.

## Conclusion

Although more thorough investigations will be required, the results from this work do offer several advances. WOB is one of the main factors in the occurrence of IPE [12, 41], but the causes of the high interindividual variability observed were not known up to now [19, 41]. Based on the results presented here, we hypothesize that the interindividual variation in WOB upon immersion is due to a similarly large variation in Crs.

Our results confirm the fundamental role of immersion-induced changes in lung function in the occurrence of IPE. Indeed, the changes to lung function (decrease in ERV, VC, and Crs, accompanied by an increase in IRV) induced by head-out-of-water immersion all significantly correlated with the number of ULCs (evidence of increased EVLW) observed after a snorkeling exercise.

Furthermore, in the face of similar immersion stresses, the extent of alterations to ventilatory function and the number of ULCs were very different between individuals but remained statistically correlated. These parameters were significantly greater in divers with a history of IPE. This variation in immersion-induced lung function impairment could be explained by individual genetic differences. Indeed, genetic makeup is known to govern the physical properties of the lungs and how they will react to immersion, thus contributing to individual susceptibility to IPE.

Alterations to pulmonary function and, in particular, to pulmonary compliance induced by head-out-of-water immersion, through their effects on WOB appear to allow the identification of divers with a greater susceptibility to developing IPE. Measurement of these parameters could therefore be proposed as a predictive test for the risk of developing IPE.

## Supplementary Information

**Additional file 1.** Individual subject values and comparative analysis of VC, ERV, and IRV and Crs.

## Data Availability

The datasets used and analyzed during the current study are available from Olivier Castagna on reasonable request.

## Abbreviations

Crs: Overall compliance of the respiratory system
ERV: Expiratory reserve volume
EVLW: Extravascular lung water
FRC: Forced residual capacity
FVC: Forced vital capacity
HAPE: High altitude pulmonary edema
IPE: Immersion pulmonary edema
IRV: Inspiratory reserve volume
ULC: Ultrasound lung comets
VC: Vital capacity
*V*_relax_: Relaxation volume
Vt: Tidal volume
WOB: Work of breathing
