# Supplementary material for: Individual Changes in Respiratory Compliance Upon Immersion May Predict Susceptibility to Immersion Pulmonary Edema
Source: Sports Med Open. 2023 Jun 1;9:39. doi: 10.1186/s40798-023-00590-8 (PMC10234985; doi:10.1186/s40798-023-00590-8)
Supplement: Supplementary file 1 — Additional file 1. Individual subject values and comparative analysis of VC, ERV, and IRV and Crs. [file 40798_2023_590_MOESM1_ESM.pdf]

Sports Medicine – Open

Manuscript Number: SMOA-D-23-00119

Individual changes in respiratory compliance upon immersion may predict susceptibility to immersion pulmonary edema.

Supplementary file

Olivier Castagna [1,2,*], Arnaud Druelle [3], Guillaume Michoud [4], Thibaut Prevautel [5], Jean-René Lacour [6]

Author Affiliations:

1. Underwater research team – ERRSO, Military biomedical research institute-IRBA, Toulon, France
2. LAMHESS (UPR 6312), Université de Nice, Nice, France
3. French Navy Diving school, St Mandrier, France
4. 2nd Regiment Etranger de Parachutistes, Calvi, France
5. Department of cardiology, Laveran military hospital (HIA Laveran), Marseille, France
6. Université Jean Monnet, 42000, Ste Etienne, France.

Corresponding author information: Pr. Olivier Castagna M.D., Ph.D. Underwater research team – ERRSO, Military biomedical research institute-IRBA, Toulon, France E-mail: castagna.olivier@gmail.com

Authors' current email addresses:

Olivier Castagna : castagna.olivier@gmail.com

Arnaud Druelle : arnaud.druelle@yahoo.fr

Guillaume Michoud : guillaume.michoud@gmail.com

Thibaut Prevautel : thibaut.prevautel@gmail.com

Jean-René Lacour : lacour.jr@wanadoo.fr

Sports Medicine – Open

Manuscript Number: SMOA-D-23-00119

Individual changes in respiratory compliance upon immersion may predict susceptibility to immersion pulmonary edema.

Supplementary file

Individual subject values for changes in spirometric data (VC, Vt, ERV, and IRV) and *Crs* induced by head-out-of-water immersion, as well as the number of ULC measured in all divers following the fin swimming exercise are reported in the two following tables.

IPE divers

Subject number	**ΔVC (L)**	**ΔERV (L)**	**ΔIRV (L)**	**ΔVt (L)**	**Δ*Crs* (L/kPa)**	**Δ*ULC***
1	-0.36	-1.56	1.06	0.14	1.86	35
2	-0.45	-1.12	0.47	0.20	0.91	17
5	-0.49	-1.56	0.90	0.17	1.39	25
6	-0.56	-2.05	1.30	0.19	1.81	33
7	-0.48	-1.80	1.13	0.18	1.51	33
9	-0.53	-1.51	0.80	0.19	1.35	18
13	-0.43	-0.77	0.10	0.24	0.68	9
15	-0.37	-1.44	0.85	0.22	1.15	19

Non-IPE divers

Subject number	**ΔVC (L)**	**ΔERV (L)**	**ΔIRV (L)**	**ΔVt (L)**	**Δ*Crs* (L/kPa)**	**Δ*ULC***
3	-0.42	-1.03	0.40	0.21	0.85	5
4	-0.41	-0.41	-0.23	0.24	0.28	0
8	-0.44	-1.15	0.50	0.21	0.94	22
10	-0.41	-0.76	0.10	0.24	0.44	0
11	-0.46	-1.41	0.74	0.21	1.12	24
12	-0.54	-1.07	0.37	0.16	0.9	11
14	-0.40	-0.81	0.16	0.25	0.71	8
16	-0.40	-0.37	-0.23	0.21	0.24	0
17	-0.54	-1.07	0.37	0.16	0.9	11
18	-0.48	-1.50	0.86	0.15	1.22	16

Vital capacity (VC); Expiratory reserve volume (ERV); Inspiratory reserve volume (IRV); Tidal volume (Vt); Ultrasound lung comets (ULC)

Individual changes in respiratory compliance upon immersion may predict susceptibility to immersion pulmonary edema.

Supplementary file

Comparative analysis of changes to VC, ERV, IRV and *Crs* induced by head-out-of-water immersion for IPE and non-IPE subjects

ΔVC

	Non-IPE	IPE
Minimum	-0.54	-0.56
25% Percentile	-0.495	-0.52
Median	-0.43	-0.465
75% Percentile	-0.4075	-0.385
Maximum	-0.4	-0.36
Mean	-0.45	-0.4588
Std. Deviation	0.05416	0.071
Std. Error of Mean	0.01713	0.0251
Lower 95% CI	-0.4887	-0.5181
Upper 95% CI	-0.4113	-0.3994
Mann Whitney test		
P value	0.7122	not significant

ΔERV

	Non-IPE	IPE
Minimum	-1.5	-2.05
25% Percentile	-1.215	-1.74
Median	-1.05	-1.535
75% Percentile	-0.6725	-1.2
Maximum	-0.37	-0.77
Mean	-0.958	-1.476
Std. Deviation	0.376	0.3921
Std. Error of Mean	0.1189	0.1386
Lower 95% CI	-1.227	-1.804
Upper 95% CI	-0.689	-1.148
Mann Whitney test		
P value	0.0079	Significant

The tables above detail the results of the respective statistical analyses corresponding to the variations in tidal volume (VC) and end reserve volume in IPE and non-IPE subjects.

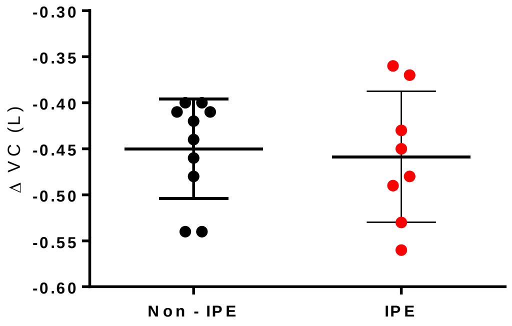

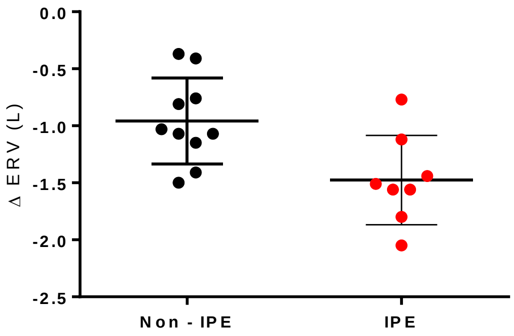

Each point corresponds to the value observed for an individual subject. Divers in the non-IPE group are represented by black dots (n=10); the red dots correspond to divers in the IPE group (n=8).

Sports Medicine – Open

Manuscript Number: SMOA-D-23-00119

Individual changes in respiratory compliance upon immersion may predict susceptibility to immersion pulmonary edema.

Supplementary file

ΔIRV

	Non-IPE	IPE
Minimum	-0.23	0.1
25% Percentile	0.0175	0.5525
Median	0.37	0.875
75% Percentile	0.56	1.113
Maximum	0.86	1.3
Mean	0.304	0.8263
Std. Deviation	0.3634	0.384
Std. Error of Mean	0.1149	0.1358
Lower 95% CI	0.04407	0.5052
Upper 95% CI	0.5639	1.147
	Mann Whitney test	
P value	0.0126	Significant

ΔCrs

	Non-IPE	IPE
Minimum	0.24	0.68
25% Percentile	0.4	0.97
Median	0.875	1.37
75% Percentile	0.985	1.735
Maximum	1.22	1.86
Mean	0.76	1.333
Std. Deviation	0.3377	0.4104
Std. Error of Mean	0.1068	0.1451
Lower 95% CI	0.5184	0.9894
Upper 95% CI	1.002	1.676
	Mann Whitney test	
P value	0.0079	Significant

The tables above detail the results of the respective statistical analyses corresponding to the Inspiratory reserve volume (IRV) and total pulmonary compliance (*Crs*) and non-IPE subjects.

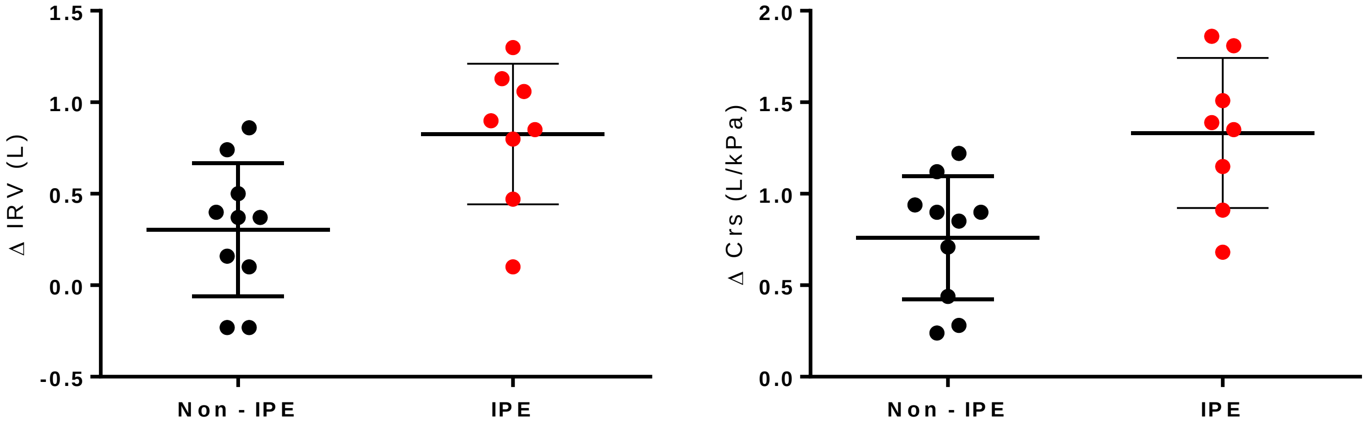

Each point corresponds to the value observed for an individual subject. Divers in the non-IPE group are represented by black dots (n=10); the red dots correspond to divers in the IPE group (n=8).
